# Mitochondrial Targeting Adaptation of the Hominoid-Specific Glutamate Dehydrogenase Driven by Positive Darwinian Selection

**DOI:** 10.1371/journal.pgen.1000150

**Published:** 2008-08-08

**Authors:** Lia Rosso, Ana C. Marques, Andreas S. Reichert, Henrik Kaessmann

**Affiliations:** 1 Center for Integrative Genomics, University of Lausanne, Lausanne, Switzerland; 2 Cluster of Excellence “Macromolecular Complexes”, Mitochondriale Biologie, Fachbereich Medizin, Johann Wolfgang Goethe–Universität Frankfurt am Main, Frankfurt am Main, Germany; University of Chicago, United States of America

## Abstract

Many new gene copies emerged by gene duplication in hominoids, but little is known with respect to their functional evolution. Glutamate dehydrogenase (GLUD) is an enzyme central to the glutamate and energy metabolism of the cell. In addition to the single, GLUD-encoding gene present in all mammals (*GLUD1*), humans and apes acquired a second *GLUD* gene (*GLUD2*) through retroduplication of *GLUD1*, which codes for an enzyme with unique, potentially brain-adapted properties. Here we show that whereas the GLUD1 parental protein localizes to mitochondria and the cytoplasm, GLUD2 is specifically targeted to mitochondria. Using evolutionary analysis and resurrected ancestral protein variants, we demonstrate that the enhanced mitochondrial targeting specificity of GLUD2 is due to a single positively selected glutamic acid-to-lysine substitution, which was fixed in the N-terminal mitochondrial targeting sequence (MTS) of GLUD2 soon after the duplication event in the hominoid ancestor ∼18–25 million years ago. This MTS substitution arose in parallel with two crucial adaptive amino acid changes in the enzyme and likely contributed to the functional adaptation of GLUD2 to the glutamate metabolism of the hominoid brain and other tissues. We suggest that rapid, selectively driven subcellular adaptation, as exemplified by GLUD2, represents a common route underlying the emergence of new gene functions.

## Author Summary

Little is known about the functional evolution of new hominoid genes. In this study, we utilized a combination of evolutionary analyses and cell biology experiments to unveil a novel mode by which the human- and ape-specific glutamate dehydrogenase enzyme (GLUD2) functionally adapted. We find that whereas the GLUD1 parental protein (present in all mammals) localizes to mitochondria and also to the cytoplasm, GLUD2 is specifically targeted to mitochondria. Using resurrected ancestral proteins and site-directed mutagenesis, we show that the optimized mitochondrial targeting capacity of GLUD2 is due to a single positively selected amino acid substitution in its N-terminal targeting sequence, which occurred soon after the duplication event in the ape ancestor 18–25 million years ago. The specialization in mitochondrial localization is probably linked to the function of GLUD2 in the glutamate metabolism of the brain (recycling of glutamate in astrocytes), but is likely also of functional relevance in other tissues in which GLUD2 is expressed. We suggest that in addition to the traditionally considered modes of functional adaptation (changes in gene expression and/or the biochemical function of the protein), rapid and selectively driven subcellular adaptation to specific ancestral compartments may represent a common yet previously little-considered mechanism for the origin of new gene functions.

## Introduction

The process of gene duplication is the major mechanism underlying the origin of new gene functions and has thus significantly contributed to the emergence of adaptive evolutionary novelties during evolution [1],[2]. DNA-based gene duplication—the duplication of chromosomal segments containing genes—has been prevalent during hominoid evolution [3]. Similarly, the process of retroposition (or “retroduplication”), a mechanism generating intronless gene copies (retrocopies) via the LINE retrotransposon-mediated reverse transcription of mRNAs from “parental” sources genes [4],[5], has resulted in a large number of gene copies in apes [6]. A small number of functional ape-specific duplicates created by these mechanisms have been identified (e.g. refs. [6]–[11]). However, although several of these genes revealed signatures of positive Darwinian selection (e.g. [9]), suggestive of adaptive protein sequence evolution, the evolutionary fate and functional protein evolution of new ape genes remains poorly understood.

GLUD2 is one of the few hominoid-specific proteins for which positively selected amino acid substitutions could be related to functional change and adaptation. It is encoded by the intronless *GLUD2* gene, which emerged via the reverse transcription of a messenger RNA from its parental gene *GLUD1* in the hominoid ancestor 18–25 million years ago (Mya) [7]. The *GLUD* genes encode two distinct isoforms of glutamate dehydrogenase (GLUD, also termed GDH), an enzyme catalyzing the oxidative deamination of glutamate to α-ketoglutarate (generating ATP through the Krebs cycle) and ammonia, a reversible reaction that takes place in mitochondria [12]. Previous work showed that the *GLUD2*-encoded enzyme evolved unique, potentially brain-specific functional properties soon after the duplication event by virtue of two key amino acid substitutions that were fixed as a result of positive selection [7],[13]. Due to these substitutions, GLUD2 shows higher activity at neutral pH than GLUD1, is less sensitive to GTP inhibition, and—unlike GLUD1—requires high ADP levels for its allosteric activation [14]. It was suggested that these properties reflect the functional adaptation of GLUD2 to the metabolism of neurotransmitter glutamate in the brain [13],[15].

Here we have further investigated the functional adaptation of the ape-specific glutamate dehydrogenase. We show that whereas GLUD1 localizes to the mitochondria as well as the cytoplasm, GLUD2 is specifically targeted to mitochondria, due to a single key amino acid substitution in its signal peptide, which emerged in the common hominoid ancestor and appears to have been fixed under the influence of positive selection. The enhanced mitochondrial targeting capacity of GLUD2 probably reflects further selectively driven optimization of this enzyme to the glutamate/energy metabolism of the brain and other tissues. More generally, the evolution of GLUD2 represents a remarkable example of rapid, selectively driven subcellular adaptation and thus reveals a novel mode for the functional adaptation of new duplicate genes.

## Results/Discussion

### Positive Selection in the Targeting Sequence of GLUD2

**Figure 1:**
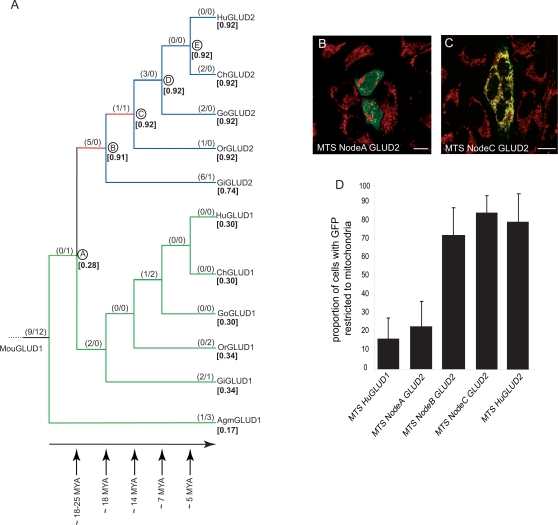
Coding Sequence Evolution of the GLUD1/GLUD2 MTSs and Subcellular Localization of MTS-GFP Fusion Proteins. (A) Phylogenetic tree based on GLUD1/GLUD2 MTS coding sequences (GLUD1 from mouse is used as an outgroup). Approximate divergence times in millions of years (MYA) are shown (estimates are based on ref. [36]). Maximum likelihood *d*_N_/*d*_S_ values and the estimated number of nonsynonymous over synonymous substitutions (in parentheses) for each branch are indicated (estimated numbers of substitutions are rounded to the nearest integer). The two internal branches for which positive selection was previously inferred for the mature protein [7] as well as here for the full-length protein (including the MTS) are highlighted in red. Overall *d*_N_/*d*_S_ rates after the duplication event for *GLUD2* (blue and red branches; *d*_N_/*d*_S_∼2) and *GLUD1* (green branches; *d*_N_/*d*_S_∼0.2) were inferred and found to be significantly different (see main text and Materials and Methods for details). Estimated probabilities of mitochondrial targeting (see Materials and Methods) are indicated within square brackets (in bold). We note that the ancestral node B and C sequences could not be unambiguously established at positions 24 and 25 due to the deletion on the gibbon lineage at these sites (the substitutions A24N and D25H were assigned to branch A–B by the reconstruction method—as depicted—although they may with equal probability have occurred on branch B–C; see Materials and Methods). Abbreviations: human CDC14Bretro, HuRetro; chimpanzee CDC14Bretro, ChRetro; gorilla CDC14Bretro, GoRetro; orang-utan CDC14Bretro, OrRetro; gibbon CDC14Bretro, GiRetro; human CDC14Bpar, HuPar. (B) LN229 cells transfected with the GLUD2 MTS (fused to GFP) from node A. (C) LN229 cells transfected with the GLUD2 MTS-GFP from node C. Mitochondria were stained (red) with MitoTracker. The images are representative of the predominant subcellular phenotypes observed for the different constructs. Only merged images are shown (co-localization of GLUD-GFP protein and mitochondria is indicated by yellow signals); unmerged images are provided in Figure S1. Scale bars = 10 µm. (D) Proportion of transfected LN229 cells in which proteins were only detected in mitochondria. Statistical analysis reveals significant subcellular localization differences between the different GLUD proteins (one-way ANOVA, *P*<10^−4^). The human GLUD1 and the node A proteins show significantly lower mitochondrial targeting capacities than the MTSs from node A, node C, or human GLUD2 (*P*<0.01, Tukey's Post Hoc test). The results of similar analyses using COS7 and HeLa cells are shown in Figure S2.

**Figure 2:**
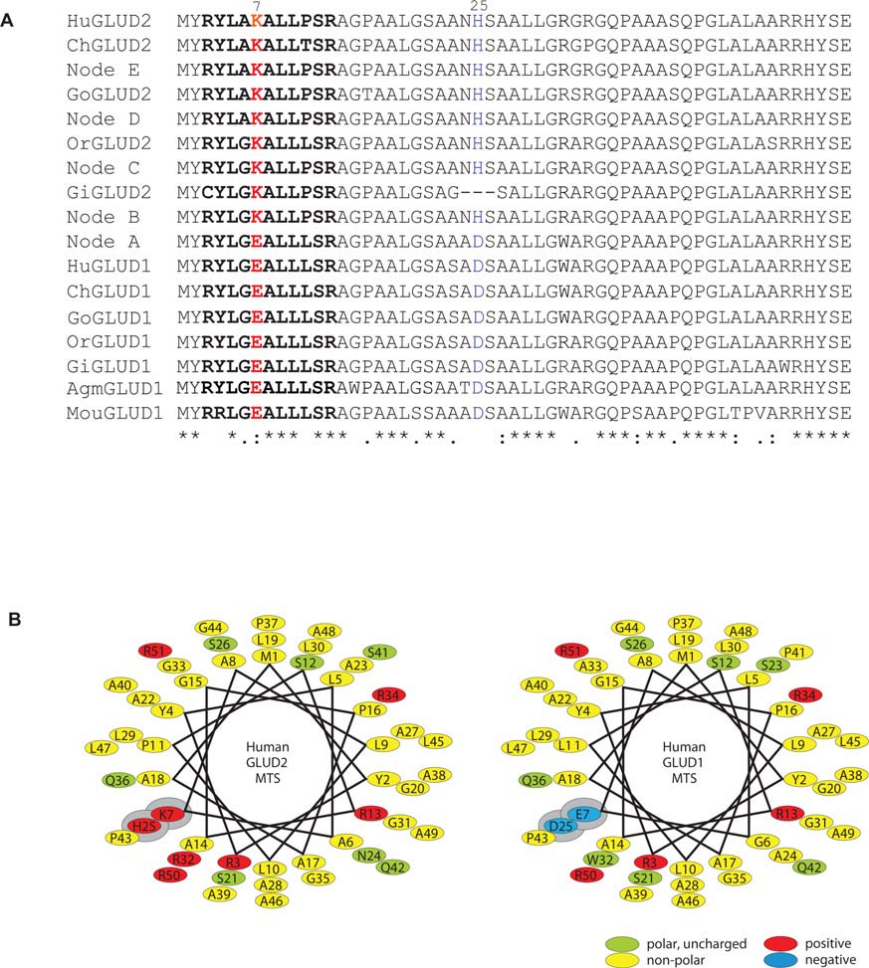
Sequences and Structures of the GLUD Mitochondrial Targeting Sequences. (A) Alignment of GLUD MTSs (same abbreviations as in Figure 1). Sites under positive selection are highlighted in bold red (residue 7) or blue (residue 25). We note that the substitutions A24N and D25H could not be unambiguously assigned to the branch node A–B and branch node B–C due to the deletion at these positions in gibbon (see also legend of Figure 1). The sequences shown were reconstructed assuming that these substitutions occurred on branch A–B. (B) Schematic representation of the MTSs of GLUD1 and GLUD2 from humans. Amino acids 1 to 51 were plotted onto a helical wheel representing an a-helix (3.6 residues per turn) viewed from the top. Polar uncharged (green), non-polar (yellow), positive (red), and negative (blue) residues are indicated. Residues predicted to be particularly important for mitochondrial targeting are highlighted in grey.

We previously identified selectively driven substitutions in the mature GLUD2 protein that led to altered enzymatic properties [7]. When further investigating the evolution of the *GLUD* coding sequences in apes, we noticed an overall significantly higher nonsynonymous to synonymous substitution rate on the *GLUD2* branches (*d*_N_/*d*_S_∼2) compared to those of *GLUD1* (*d*_N_/*d*_S_∼0.2; *P*<10^−3^) after the duplication event, when restricting the analysis to the 5′-end (first 159 nucleotides) of the sequences (Figure 1A, see also legend and Materials and Methods for details). Notably, this region codes for a mitochondrial targeting sequence (MTS) of 53 amino acids (Figure 2A), which is required to direct the GLUD1 enzyme to mitochondria [16].

Prompted by this observation, we sought to assess whether the accelerated change of the MTS of GLUD2 reflects the action of positive selection (rather than relaxation of selective constraint) and might therefore be of functional relevance. To this end, we traced the evolutionary history of the full-length *GLUD2* coding sequence (including the MTS-encoding sequence), focusing on the first two internal branches after the duplication event (Figure 1A). These branches were previously shown to represent an adaptive phase during the evolution of the mature GLUD2 protein [7]. A maximum likelihood procedure that tests for selection at certain sites (see Materials and Methods for details) revealed two amino acid substitutions (position 7 and 25) under positive selection in the MTS region (*P*>0.95, Figure 2A). We thus hypothesized that GLUD2 might have functionally adapted by evolving altered subcellular targeting.

### GLUD2 Is Specifically Targeted to Mitochondria

To explore this hypothesis, we first used an *in silico* method ([17], Materials and Methods) to predict subcellular localization of reconstructed ancestral GLUD2 MTS variants, representing sequences before (sequence upon duplication event, node A) and after (great ape ancestor, node C) the period of positive selection (Figure 1A). Interestingly, this analysis suggested a substantially higher mitochondrial targeting probability for the node C targeting sequence (0.92) than for that of node A (0.28; Figure 1A).

To experimentally test these predictions, we synthesized the reconstructed MTSs for the node A and node C variants and fused them to a fluorescent (GFP) reporter. As GLUD2 is thought to have particularly adapted to a function in degrading neurotransmitter glutamate in astrocytes [13],[14], we transfected a human astrocyte-derived cell line (LN229, glioblastoma) with a vector encoding these recombinant proteins. These experiments revealed a striking pattern. We found that the node A MTS-GFP fusion protein localized to mitochondria, as expected, but that it could also be detected in the cytoplasm in most cells (Figure 1B, 1D, and Figure S1). In contrast, the node C MTS protein localized specifically to mitochondria in the vast majority of cells (Figure 1C, 1D, and Figure S1). Thus, consistent with the *in silico* predictions, our experimental analysis strongly suggests that the MTS of GLUD2 evolved the capacity to more specifically target the GLUD2 enzyme to mitochondria during the period of positive selection.

Further localization experiments showed that—similarly to the node A protein (Figure 1B, 1D, and Figure S1)—the human GLUD1 MTS-GFP fusion protein generally localizes to both mitochondria and the cytoplasm (Figure 1D and Figure S1). This suggests that GLUD1 preserved the ancestral localization pattern since the time of the duplication event (node A), which is consistent with the paucity of amino acid substitutions during GLUD1 evolution and its low mitochondrial targeting prediction value (0.30, Figure 1A).

These experiments also showed that the human GLUD2 MTS retained the increased mitochondrial targeting capacity (Figure 1D and Figure S1), in agreement with the high mitochondrial localization probability (0.92) estimated *in silico* (Figure 1A). Thus, the enhanced mitochondrial targeting specificity of the GLUD2 MTS was preserved after the period of positive selection on the lineage leading to humans. We obtained similar results for two other cell lines (human HeLa cells and COS7 from African green monkeys), further supporting the notion of a subcellular targeting shift of GLUD2 during its early evolution (Figure S2).

To more precisely date the shift of the GLUD2 targeting specificity, we assessed the subcellular localization of GLUD2 from the last common hominoid ancestor (node B). We found that the resurrected node B protein localized specifically to mitochondria in the majority of cells (Figure 1D and Figure S1), consistent with the high mitochondrial prediction value (0.91, Figure 1A). This suggests that GLUD2 had already evolved an increased mitochondrial localization specificity in the common hominoid ancestor ∼18–25 million years ago.

**Figure 3:**
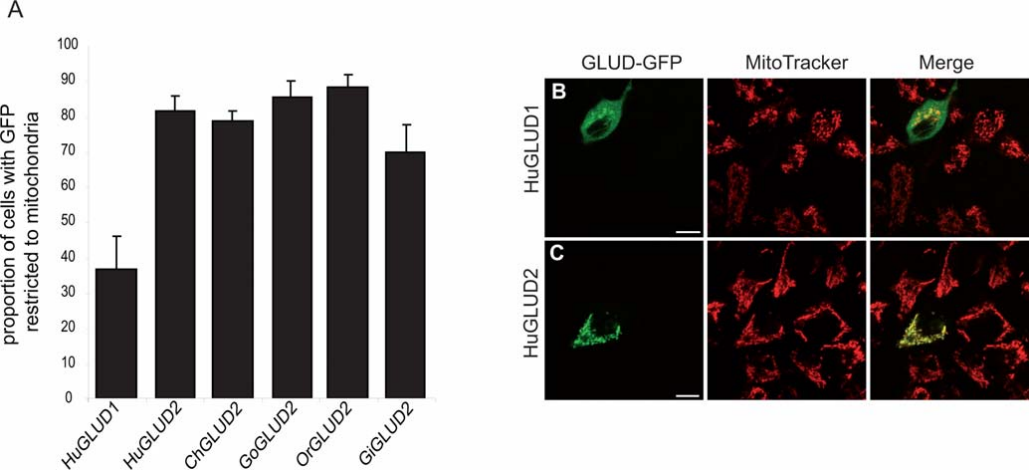
Subcellular Localization of GLUD2 from Hominoids and Human GLUD1. (A) Proportion of transfected HeLa cells in which GFP signals from the different recombinant GLUD-GFP fusion proteins were only detected in mitochondria. Human GLUD1 shows significantly lower mitochondrial localization specificities than GLUD2 from any of the apes (*P*<0.01, Tukey's Post Hoc test). GLUD2 from gibbons shows a lower mean mitochondrial targeting capacity in the experiments than GLUD2 from the other apes (which might indicate that some of the enhanced targeting capacity was lost on the gibbon lineage), although this difference is only statistically significant when compared to GoGLUD2 and OrGLUD2 (*P*<0.05, Tukey's Post Hoc test). We note that LN229 and COS7 cells could not be analyzed, as these cell lines do not tolerate expression of additional recombinant glutamate dehydrogenase (presumably due to the higher expression levels of recombinant GLUD proteins in these cells upon transfection). (B) and (C) HeLa cells transfected with human GLUD1 and GLUD2 GFP fusion constructs, respectively (mitochondria labeled with MitoTracker, red). Scale bars = 10 µm. Unmerged images are shown in Figure S3.

To assess the subcellular targeting capacities of the GLUD MTS sequences in the context of their physiologically targeted proteins, we performed similar experiments using full-length GLUD-fluorescent protein fusions. These experiments confirm the results obtained using the MTS-GFP fusions for the human GLUD1 and GLUD2 proteins (Figure 3A–C and Figure S3). We also analyzed the subcellular localization of extant GLUD2 proteins from the other apes. Indeed, GLUD2 from all apes localizes predominantly to mitochondria (Figure 3A and Figure S3). Thus, the enhanced mitochondrial targeting specificity of GLUD2 was conserved throughout hominoid evolution.

### Enhanced Mitochondrial Targeting through Single Amino Acid Substitution in the Ape Ancestor

Which substitutions in the GLUD2 MTS contributed to the increased mitochondrial targeting capacity? A typical MTS contains several positively charged residues, such as lysines or arginines, and hydrophobic residues, generating an amphipathic helix [18],[19]. Due to the electrical potential across the inner membrane of mitochondria (the mitochondrial matrix being negatively charged), positive charges within the MTS are assumed to electrophoretically promote transfer of proteins across this membrane [18],[19].

One of the two positively selected amino acid changes in the GLUD2 MTS involves a glutamic acid (E) to lysine (K) substitution at position 7 of the sequence (Figure 2A). Notably, this substitution–which occurred in the common hominoid ancestor during the time of the switch in targeting specificity–introduces a positive charge to the MTS by replacing a negatively charged residue (Figure 2A). The second amino acid substitution under positive selection (D25H) replaces a negatively charged residue (aspartate, D) and at the same time introduces a partially positively charged amino acid (histidine, H) at position 25 of the MTS.

A helical wheel representation of the secondary structure of the helix formed by the GLUD2 MTS illustrates its modified properties (Figure 2B). The E7K and D25H substitutions introduce additional positively charged amino acids at one side of the α-helix within a previously weakly positively charged patch. Opposite to this charged patch, an ancestral patch with hydrophobic amino acids is found, which has remained largely unchanged during the evolution of GLUD2. Thus, the two positively selected substitutions are predicted to promote the formation of an amphipathic helix, which may function as an optimized MTS.

Consistently, changing these residues in the different GLUD sequences alters the *in silico* predictions of the GLUD mitochondrial targeting capacities. In particular, the E7K substitution dramatically alters these predictions. For example, introducing this substitution into the human GLUD1 sequence leads to an increase of the mitochondrial targeting probability from 30% to 90%, whereas the D25H substitution increases the GLUD1 wild type value to only 35%. The predominant effect of E7K is expected, given that this substitution represents the more radical substitution, as it replaces a negatively charged residue with a fully positively charged amino acid (see above, Figure 2B). Thus, we hypothesized that the E7K substitution was the key contributor to the evolution of optimized mitochondrial targeting of GLUD2.

**Figure 4:**
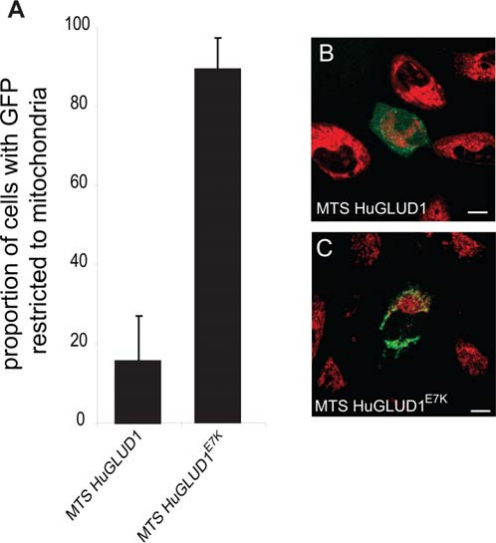
Subcellular Localization of Wild-Type and Mutant GLUD1 MTS. (A) Proportion of transfected LN229 cells with GLUD-GFP signals detected only in mitochondria. HuGLUD1^E7K^ shows significantly higher mitochondrial localization specificity than HuGLUD1 (*P*<0.01, Tukey's Post Hoc test). (B) and (C) LN229 cells transfected with GLUD1 and GLUD1^E7K^ GFP fusion constructs, respectively (mitochondria labeled with MitoTracker, red). Scale bars = 10 µm. Unmerged images are shown in Figure S4.

**Figure 5:**
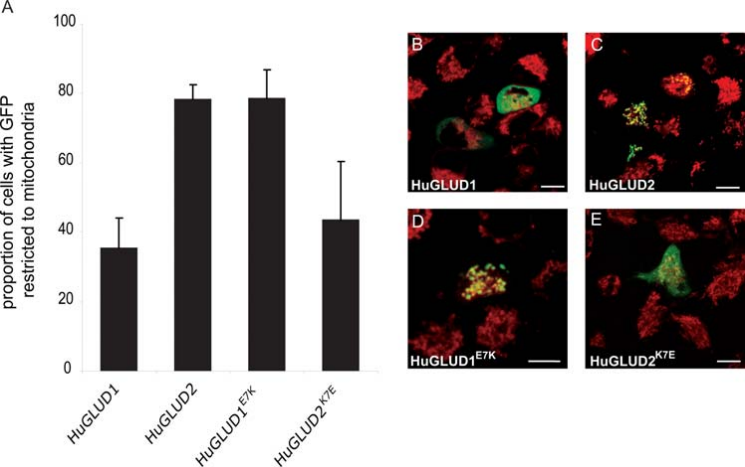
Subcellular Localization of GLUD1 and GLUD2 Proteins With Wild Type and Mutant MTSs, respectively. (A) Proportion of transfected HeLa cells with GLUD-GFP signals detected only in mitochondria. HuGLUD2^K7E^ shows significantly lower mitochondrial localization specificity than HuGLUD2 and HuGLUD1^E7K^ (*P*<0.01, Tukey's Post Hoc test). (B)–(E) HeLa cells transfected with wild type and mutant GLUD proteins (mitochondria labeled with MitoTracker, red). Scale bars = 10 µm. Unmerged images are shown in Figure S5.

To test this hypothesis, we introduced the E7K substitution into the MTS of human GLUD1 using site-directed mutagenesis. Remarkably, the mutant GLUD1^E7K^ MTS shows a dramatic increase in mitochondrial localization capacity relative to the wild type variant (Figure 4), which is indistinguishable from those observed for extant or ancestral GLUD2 variants from node B and C (Figure 1 and Figure S1). We obtained similar results when introducing the E7K substitution into the full-length GLUD1 protein (Figure 5). Thus, in accord with our prediction, the subcellular adaptation of GLUD2 appears to have been mainly driven by this one key substitution that occurred soon after the retroduplication event in the common hominoid ancestor.

In support of the notion that E7K substitution was key to the increased mitochondrial targeting capacity of GLUD2, we find that reverting this substitution back to the ancestral glutamic acid in the GLUD2 sequence reduces its mitochondrial targeting specificity to a level that is indistinguishable from that of the parental GLUD1 protein (Figure 5). In conclusion, while the D25H substitution and potentially other substitutions that occurred during the period of positive selection might have contributed to enhanced or altered mitochondrial targeting *in vivo*, the key substitution rendering GLUD2 specific to mitochondria was E7K. Notably, this residue is conserved (as glutamic acid) in GLUD targeting sequences from other mammals, including mouse (Figure 2A) and opossum (not shown), a marsupial that diverged from primates around 180 Mya [20]. Generally, our results lend striking experimental support to a hypothesis suggesting that subcellular localization changes of duplicate proteins could occur by key substitutions in protein targeting sequences [21].

We finally note that the GLUD2 MTS seems to have lost some of the enhanced mitochondrial targeting specificity on the gibbon lineage (Figure 3), consistent with the lower *in silico* prediction value for the gibbon GLUD2 MTS (0.74, Figure 1A). This is presumably mainly due to a substitution at the third position of the gibbon GLUD2 MTS–a change from the ancestral arginine residue in the positively charged patch of the MTS helix to a non-charged cysteine residue (Figure 2A)–which reduces the net positive charge of the protein and leads to a reduction of the *in silico*-predicted mitochondrial localization probability (from 0.92 to 0.74).

### Potential Functional Implications of the Subcellular Adaptation of GLUD2

Based on previous work, it was suggested that the emergence of *GLUD2* in hominoids may have permitted an increased astrocyte metabolism of glutamate [7],[13]. GLUD2 evolved its unique enzymatic properties soon after the duplication event in the common hominoid ancestor (∼18–25 Mya), on the basis of two positively selected amino acid substitutions in the mature protein (see introductory paragraph and refs. [7],[13],[14]).

Here we have identified an additional mechanism through which GLUD2 appears to have functionally adapted. We show that GLUD2 evolved an enhanced mitochondrial targeting specificity, mainly by virtue of a single amino acid change in its MTS, which also appeared during the period of positive selection in the common hominoid ancestor. Thus, while its parental protein GLUD1 localizes to mitochondria (as previously reported, ref. [16]) but also to the cytoplasm, the subcellular localization of GLUD2 is largely restricted to mitochondria.

What was the selective benefit of the enhanced mitochondrial targeting capacity of GLUD2? We propose two—not mutually exclusive—scenarios that may explain this observation. First, in addition to its mitochondrial function, *GLUD1*-encoded GDH may have an–as yet–unknown function in the cytoplasm, akin to other mitochondrial enzymes (e.g., fumarase, ref. [22]). Due to the changes in its targeting sequence upon the retroduplication event, the ape-specific GLUD2 protein evolved a specific function in one of these ancestral compartments–the mitochondrion. This subcellular adaptation might have been particularly relevant with respect to the presumed function of GLUD2 in astrocytes (see above), where it is thought to be involved in the degradation/metabolism of neurotransmitter glutamate—a process taking place in mitochondria. We note, however, that recent work revealed that *GLUD2* (similarly to *GLUD1*) is transcribed in many or most human tissues (Bryk et al., unpublished). This finding is in contrast to a previous study, which suggested that *GLUD2* is rather specifically expressed in brain, retina, and testis [23]. Consequently, the subcellular adaptation of GLUD2 is likely of functional significance for hominoid tissues in general.

A second possibility that might explain the more specific mitochondrial targeting of GLUD2 involves the rather large variability of mitochondrial membrane potentials, which depend on the tissue and cell type [24],[25]. While mitochondria in tissues such as heart and muscle have high membrane potentials (i.e., they are more negatively charged inside the mitochondrial matrix than mitochondria in cells from other tissues), glial cells—such as astrocytes—have lower membrane potentials. Thus, GLUD2 may have evolved a more positively charged targeting sequence to compensate for the low membrane potential of mitochondria in astrocytes, thus ensuring efficient import of *GLUD2*-encoded GDH into mitochondria in these glial brain cells.

Further work is required to more precisely understand the physiological implications of the enhanced mitochondrial localization specificity of this recently emerged hominoid protein. In any event, our results suggest that the shift in subcellular targeting specificity of GLUD2 was beneficial to the evolution of the glutamate/energy metabolism of the hominoid brain and/or other tissues, as it appears to have been driven by positive selection.

More generally, our study provides a remarkable example of a novel mode for the origin of new gene functions [21], [26]–[28]. It has long been known that paralogous protein family members may localize differently in the cell (e.g., ref. [29]). Indeed, recent work using yeast as a model system suggests that subcellular adaptation represents a rather common mechanism through which duplicate genes may functionally diversify [30]. Interestingly, a hominoid-specific protein was recently shown to have completely changed its subcellular localization during its evolution due to positive selection [31], thus representing a case of “neolocalization” [30]. Here we have shown that newly emerged proteins such as GLUD2 may rapidly adapt to specific ancestral compartments (a process termed “sublocalization”; ref. [30]) under the influence of positive selection at key sites. We thus suggest that in addition to changes in gene expression and/or the biochemical function of the protein, rapid and selectively driven subcellular adaptation (by either neo- or sublocalization) is likely to represent a common mechanism underlying the emergence of new gene function.

## Materials and Methods

### Evolutionary Analysis

The phylogenetic tree of the *GLUD1/GLUD2* sequences coding for the mitochondrial targeting peptide was based on the known *GLUD* topology (ref. [7], which also corresponds the commonly accepted hominoid species phylogeny). *d*_N_/*d*_S_ ratios and the number of synonymous and nonsynonymous changes in the phylogenetic tree were estimated using the codeml free-ratio model as implemented in the PAML4 package [32]. To assess whether the *d*_N_/*d*_S_ ratio of the *GLUD2* MTS is significantly elevated relative to that of *GLUD1*, we first compared a one-ratio codeml model (which assumes an equal *d*_N_/*d*_S_ ratio for all the branches in the phylogeny) to a two-ratio model, where an additional *d*_N_/*d*_S_ ratio is allowed on the *GLUD2* lineages. Differences between these two models as well as the null and alternative models described in the following were compared using a likelihood-ratio test [33]. We note that the *d*_N_/*d*_S_ rate of *GLUD1* after the duplication event is not significantly different from that in the remaining *GLUD1* branches in the tree (*P*<0.49), which suggests that the selective constraint on the coding sequence of *GLUD1* has not changed after the emergence of *GLUD2*.

To assess whether the *GLUD2* coding sequence (including its MTS) has evolved under the influence of positive selection, we used a conservative branch-site test [34]. We compared the likelihood of a model, which allows for *d*_N_/*d*_S_>1 at a subset of sites (i.e., *d*_N_/*d*_S_ is estimated from the data) on the two internal branches after the duplication event, to that of a null model where *d*_N_/*d*_S_ of this site class was fixed to 1. The *d*_N_/*d*_S_ ratio was found to be significantly larger than 1 (*P*<0.02), consistent with a previous analysis focusing on the sequence encoding the mature protein [7].

Specific sites under positive selection were predicted using a Bayesian approach [35] as implemented in codeml. The ancestral sequences for nodes A, B, and C, were reconstructed using a one-ratio model (M0) as implemented in codeml. The posterior probabilities for reconstructed codons at all nodes were high (>0.95). Only the ancestral sequences for the two codons at positions 24 and 25 could not be unambiguously determined at nodes B and C, as these positions overlap with the deletion of 9 nucleotides in gibbon and two substitutions occurred at these positions on the branches between nodes A and C. The substitutions were assigned to branch A–B (Figure 1A and 2A), as determined by codeml, but could equally be assigned to branch B–C.

### Targeting Peptide Screen and Subcellular Localization Prediction

To analyze the mitochondrial targeting sequences of GLUD1 and GLUD2 and to assess subcellular localization, we used the PREDOTAR software ([17], http://urgi.versailles.inra.fr/predotar/french.html). We note that other target sequence analysis/subcellular prediction tools provided similar results (not shown).

### Structural Analysis of Targeting Sequences

To analyze the structure and property changes of the GLUD1/GLUD2 mitochondrial targeting sequences, we used a helical wheel prediction tool (http://rzlab.ucr.edu/scripts/wheel/wheel.cgi).

### Recombinant Proteins

GLUD1 and GLUD2 coding sequences were obtained by PCR (primers sequences available upon request) using the following primate genomic DNA samples from the ECACC repository (Wiltshire, UK): Human “Caucasian”, chimpanzee (*Pan troglodytes*), gorilla (*Gorilla gorilla*), orangutan (*Pongo pygmaeus*), gibbon/siamang (*Symphalangus syndactylus*), and African green monkey (*Cercopithecus aethiops sabaeus*). The reconstructed GLUD sequences (see above, section Evolutionary Analysis) were synthesized by GenScript and cloned. GLUD targeting sequence mutants were obtained through site-directed mutagenesis by introducing the substitutions E7K and K7E in the GLUD1 and the GLUD2 sequences, respectively (all primers and restriction enzymes used are available upon request). All sequences were cloned into pEGFP-N1 (Clontech) vectors using standard procedures.

### *GLUD* Sequences

*GLUD* sequences that were not already available (*GLUD1* MTS coding sequences from apes and African green monkey) were determined using standard sequencing procedures (sequences were run on an ABI 3730 automated sequencer) and the samples described above. These sequences were deposited in Genbank (see below for accession numbers).

### Subcellular Localization Experiments

HeLa, LN229 and COS7 cells were cultivated under standard conditions. Cells grown on MatTek Glass Bottom Culture Dishes (MatTek) for 24 hours were transiently transfected with the different *GLUD* constructs using Lipofectamine Plus (Invitrogen) according to the protocol of the supplier. 23.5 hours after transfection, mitochondria were stained with MitoTracker Red CMXRos (Invitrogen). Living cells were analyzed using a Confocal Microscope Zeiss LSM 510 Meta INVERTED by using a 63-fold oil objective. We used LSM for image analysis. In order to quantify the number of transfected cells that express GLUD proteins specifically in mitochondria, or in both the cytoplasm and mitochondria, we assigned a code to each dish with the respect to the construct used for transfection. We then proceeded with blind counts of the cellular phenotypes for each experiment. Specifically, the percentage of cells with GFP signals only in mitochondria was assessed by examining 10–50 transfected cells at 40-fold magnification over ten arbitrarily chosen areas on the dish. Each experiment was repeated five times. Differences between treatment groups were evaluated using ANOVA followed by a Post Hoc (Tukey HSD Test), with significance set at *P*<0.01.

### Accession Numbers

The Genbank (http://www.ncbi.nlm.nih.gov/Genbank/) accession numbers for the previously unpublished *GLUD1* MTS coding sequences are: EU828516 (chimpanzee, *Pan troglodytes*), EU828520 (gorilla, *Gorilla gorilla*), EU828517 (orang-utan, *Pongo Pygmaeus*), EU828518 (Siamang, *Symphalangus syndactylus*), and EU828519 (African green monkey, *Cercopithecus aethiops sabaeus*).

## Supporting Information

Figure S1 Subcellular Localization of GLUD MTS-GFP Fusion Proteins. See legend of Figure 1 and main text for details.
(8.55 MB TIF)

Figure S2 Proportion of Cells With Localization of GLUD1/GLUD2 MTS-GFP Proteins Restricted to Mitochondria. (A) COS7 cells. (B) HeLa cells.
(0.42 MB EPS)

Figure S3 Subcellular Localization of Human GLUD1 and GLUD2 from Apes. See legend of Figure 3 and main text for details.
(9.63 MB TIF)

Figure S4 Subcellular Localization of Wild Type and Mutant GLUD MTS-GFP Fusion Proteins. See legend of Figure 4 and main text for details.
(5.03 MB EPS)

Figure S5 Subcellular Localization of Wild Type and Mutant GLUD1/GLUD2 MTS-GFP Fusion Proteins. See legend of Figure 5 and main text for details.
(10.21 MB EPS)
