# Supplementary figures and images for: Mitochondrial Targeting Adaptation of the Hominoid-Specific Glutamate Dehydrogenase Driven by Positive Darwinian Selection
Source: PLoS Genet. 2008 Aug 8;4(8):e1000150. doi: 10.1371/journal.pgen.1000150 (PMC2478720; doi:10.1371/journal.pgen.1000150)

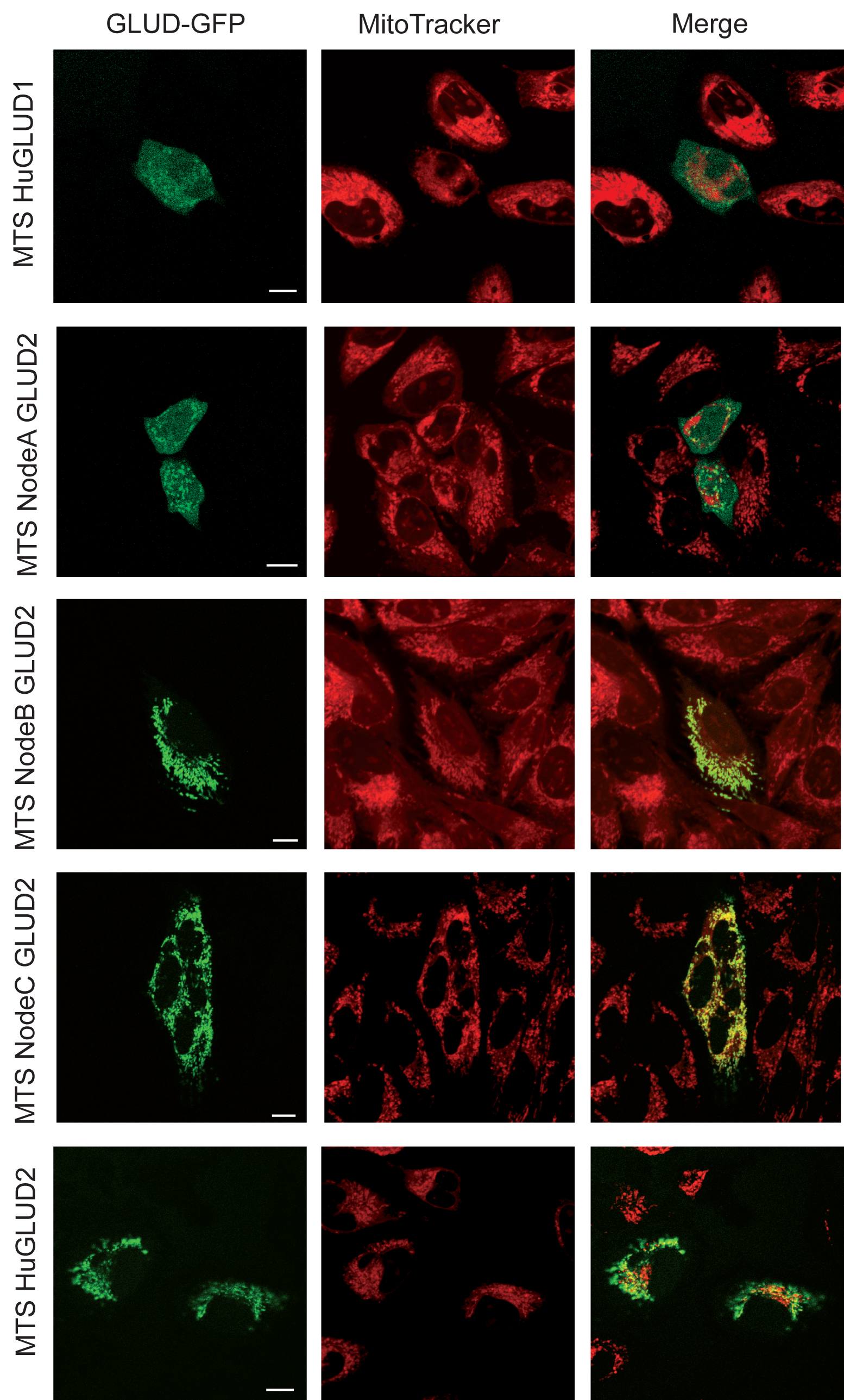

Supplement: Figure S1 — Subcellular Localization of GLUD MTS-GFP Fusion Proteins. See legend of Figure 1 and main text for details. (8.55 MB TIF) [file pgen.1000150.s001.tif]

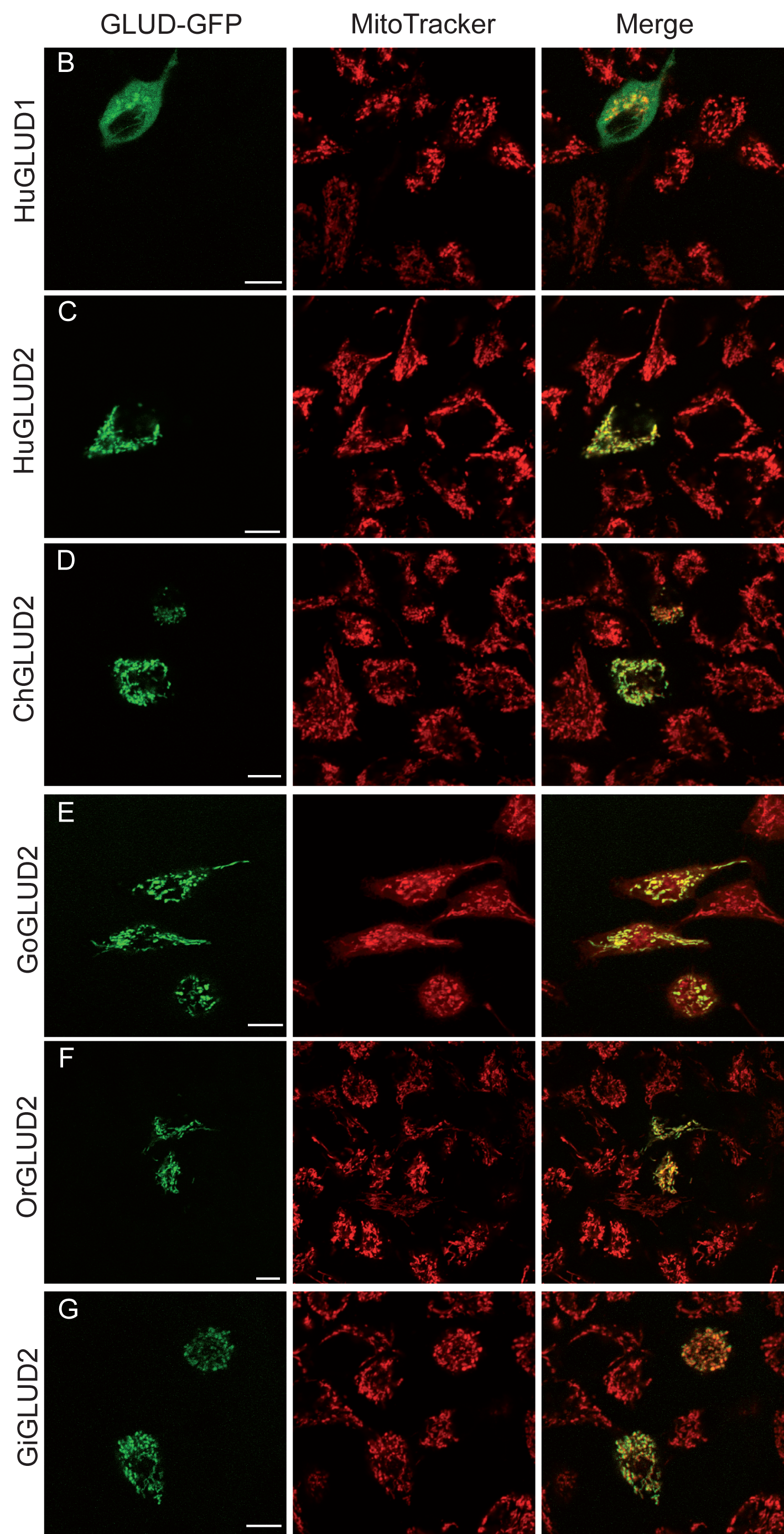

Supplement: Figure S3 — Subcellular Localization of Human GLUD1 and GLUD2 from Apes. See legend of Figure 3 and main text for details. (9.63 MB TIF) [file pgen.1000150.s003.tif]
